# Maternal hepatitis B surface antigen carrier status and pregnancy outcome: a retrospective cohort study

**DOI:** 10.1017/S0950268822000681

**Published:** 2022-04-20

**Authors:** Yiming Chen, Wenwen Ning, Xue Wang, Yijie Chen, Bin Wu, Jie Tao

**Affiliations:** 1 Department of Prenatal Diagnosis and Screening Center, Hangzhou Women's Hospital (Hangzhou Maternity and Child Health Care Hospital), Hangzhou, Zhejiang 310008, China; 2 Department of the Fourth School of Clinical Medical, Zhejiang Chinese Medical University, Hangzhou, Zhejiang 310053, China; 3 Department of Reproduction Center, Xuzhou Maternity and Child Health Care Hospital, Xuzhou, Jiangsu 221010, China; 4 Department of Science and Education, Hangzhou Women's Hospital (Hangzhou Maternity and Child Health Care Hospital), Hangzhou, Zhejiang 310008, China

**Keywords:** Carrier, hepatitis B surface antigen, intrahepatic cholestasis of pregnancy, pregnancy outcome, thrombocytopenia

## Abstract

To investigate the effect of maternal hepatitis B surface antigen (HBsAg) carrier status during pregnancy on pregnancy outcomes in a population of patients in Hangzhou, China. A retrospective cohort study was conducted to analyse data from 20 753 pregnant women who delivered at Hangzhou Women's Hospital between January 2015 and March 2020. Of these, 18 693 were normal pregnant women (the non-exposed group) and 735 were HBsAg carriers (the exposed group). We then analysed by binary multivariate logistic regression to determine the association between maternal HBsAg-positive and adverse pregnancy outcomes. The prevalence of HBsAg carriers was 3.78% and the odds ratio (OR) for maternal age in the exposed group was 1.081. Pregnant women who are HBsAg-positive in Hangzhou, China, are at higher risk of a range of adverse pregnancy outcomes, including intrahepatic cholestasis of pregnancy (ICP) (adjusted OR (aOR) 3.169), low birth weight (aOR 2.337), thrombocytopenia (aOR 2.226), fallopian cysts (aOR 1.610), caesarean scar pregnancy (aOR 1.283), foetal distress (aOR 1.414). Therefore, the obstetricians should pay particular attention to ICP, low birth weight, thrombocytopenia, fallopian cysts, caesarean scar, foetal distress in HBsAg-positive pregnant women.

## Introduction

A hepatitis B surface antigen (HBsAg) carrier is defined as a patient with serum HBsAg positivity, HBeAg negativity, HBV DNA levels below the lower limit of detection and a concentration of alanine aminotransferase that lies within the normal range on three or more consecutive follow-up visits within 1 year, each separated by at least 3 months. Histological examination of the livers of HBsAg-positive women exhibits a histological activity index score <4 points and lesions that were judged to be mild according to other semi-quantitative scoring systems [1].

According to the 2017 World Health Organization (WHO) Global Hepatitis Report, approximately 292 million people around the world were carriers of the hepatitis B virus in 2016, thus representing approximately 4% of the global population [2, 3]; of these, the HBV seroprevalence in pregnant women was 8% [4, 5]. The prevalence of HBV infection among birthing women throughout the USA was reported to be 85.8/100000 cases while the rate of maternal HBV infection has increased by 5.5% annually over recent years [6]. In Africa, however, pregnancy-related hepatitis B occurs in 14% of cases during pregnancy (95% confidence interval (CI) 10–18%) and 16% (95% CI 11–24%) after delivery [7]. In a previous study, Akhter *et al*. [8] showed that intrapartum transmission is the major mode of vertical transmission, and irrespective of HBeAg status; in contrast, the rate of transmission was almost 100% for mothers who were HBeAg-positive.

There are few reports in the existing literature that describe whether HBsAg carriers are associated with adverse pregnancy outcomes. In a previous study, Tan *et al*. [9] suggested that HBsAg positivity during pregnancy was associated with a higher risk of multiple adverse outcomes for the mothers, and that pregnant women who were HBsAg-positive were at higher risk of developing gestational diabetes mellitus (GDM), postpartum haemorrhage (PPH), intrahepatic cholestasis of pregnancy (ICP) and caesarean section. In another study, Wu *et al*. [10] reported that HBsAg-positive pregnant women may be at an increased risk of GDM, ICP, preterm delivery and neonatal asphyxia. Therefore, it is evident that obstetricians should pay particular attention to ICP, PPH, placental abruption and preterm delivery, in HBV-positive pregnant women [11]. However, Wong *et al*. [12] showed that HBsAg positivity in pregnant women does not pose an additional risk to pregnancy. Chen *et al*. [13] also suggested that an HBsAg carrier does not increase the risk of adverse neonatal outcomes or child growth and that enhanced surveillance for adverse neonatal complications in HBV-infected pregnant women may not be necessary.

Therefore, we aimed to investigate the effect of an HBsAg carrier status during pregnancy on pregnancy outcomes in a population of subjects from Hangzhou, China. This was a retrospective cohort study and included data from 19 428 pregnant women, including 735 HBsAg carrier status, for risk factor analysis.

## Materials and methods

### Subjects

**Fig. 1.**
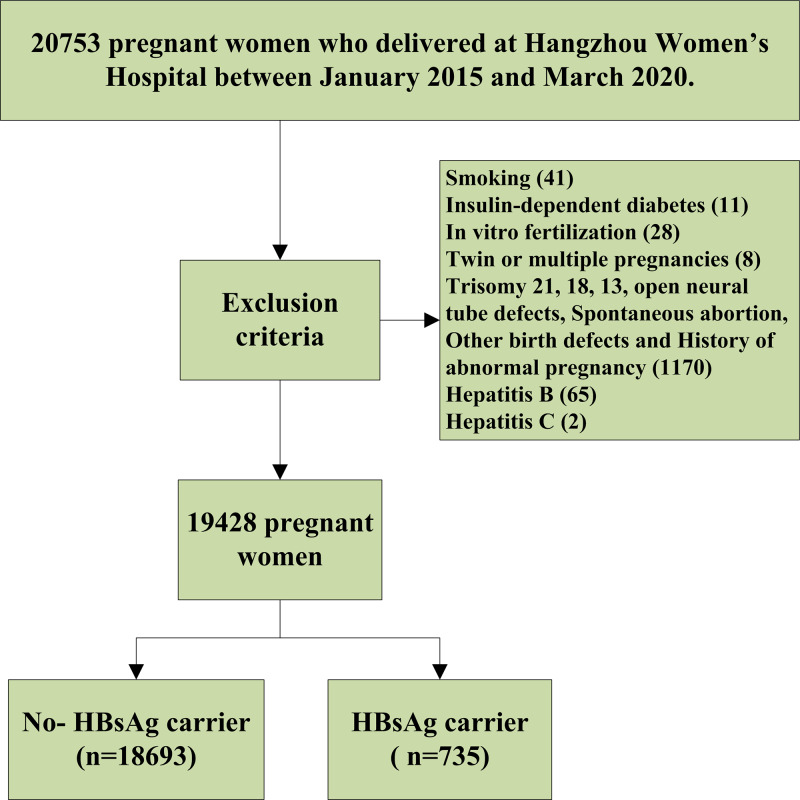
Flowchart for this study.

Using a retrospective cohort design, we collected data from 20 753 pregnant women who delivered in the Department of Obstetrics at Hangzhou Women's Hospital between January 2015 and March 2020. Of them, 1325 cases were excluded after using the inclusion criteria, as shown in Figure 1. Of these, 18 693 were normal pregnant women (the non-exposed group) and 735 were pregnant women with HBsAg carrier status (the exposed group). All pregnant women were tested for hepatitis B triad during the first prenatal examination between 11 and 13 weeks of gestation, and HBV-DNA test was performed for HBsAg-positive patients. The status of HBsAg-positive patients was recorded in the patient's medical records. Every pregnant woman receives a health record card (health number) with a unique number at the time of her first pregnancy and all data (including medical examination, laboratory examination, medical treatment and doctor's orders) are recorded at the hospital. All of the study subjects were singleton pregnancies. All pregnant women are followed up in these hospitals until delivery. This study was approved by the medical ethics committee of the Hangzhou Women's Hospital ([2022] Medical Ethics Review K (3)-05).

### Diagnostic and exclusion criteria

#### Diagnostic criteria

The diagnosis was made according to the requirements of the guideline of prevention and treatment for chronic hepatitis B: a 2015 up-date by the Chinese Society of Hepatology of the Chinese Medical Association [1], and according to serological, virological and biochemical criteria, and other clinical and auxiliary examinations of HBV-infected individuals.

#### Exclusion criteria

Pregnant women were excluded from the study if they are involved with twin or multiple pregnancies, smoking, spontaneous abortion or *in vitro* fertilisation. Patients were also excluded if the follow-up results revealed trisomy 21, 18, 13 open neural tube defects, and other birth defects; if there was a history of immunotherapy and blood transfusion; a history of special medications during pregnancy; hepatitis B, C infection; a history of pre-GDM, chronic hypertension and complications associated with chronic hypertension such as heart disease, kidney disease, connective tissue disease and haematological disease. Patients were also excluded if any data were missing.

### Pregnancy complications and pregnancy outcomes

Pregnancy complications included hypertensive disorder pregnancy, GDM, ICP (pruritus) and serum total bile acid (≥10 μmol/l), thrombocytopenia (platelet count of <100 × 10/μl coupled with at least a 25% drop from the baseline count), fallopian cysts, caesarean scar (the scar from the last caesarean section). Pregnancy outcomes included premature rupture of membranes (PROM), foetal distress, preterm delivery, low birth weight (<2500 g), macrosomia (≥4000 g) and PPH (blood loss of ≥500 ml for vaginal delivery, ≥1000 ml for caesarean section). All pregnancy complications and pregnancy outcomes were obtained from clinical records and were diagnosed by obstetricians in hospitals according to the corresponding Chinese guidelines [14–17].

### Statistical analysis

Statistical analyses were performed using IBM-SPSS version 24.0 Statistics (IBM-SPSS, Chicago, USA). Univariate analysis was performed on qualitative data by Pearson's *χ*^2^ test or the continuity correction B test. Binary multivariate logistic regression analysis was used to determine the odds ratio (OR) and 95% CIs of variables relating to each relevant influencing factor. After adjusting potential confounding variables (Backward: LR: at the basis of including all candidate variables, remove the independent variables that do not meet the significant level of retention requirements from the model at a time, and then the variables left would enter the model), the maternal HBsAg positive status on pregnancy outcomes was performed by adjusted odds ratios (aOR). Variables in step 1 were maternal age, ICP, thrombocytopenia, cesarean scar, fallopian cysts, fetal distress, cesarean delivery, low birth weight. *P* < 0.05 was considered to be statistically significant.

## Results

### Basic demographic data

Of the 19 428 pregnant women included in this study, we identified 735 maternal HBsAg carrier statuses (the exposure group); the incidence of maternal HBsAg carriers was 3.78%. The mean maternal age of the HBsAg carrier group was 29.72 (23.94–34.91) years; this was significantly higher than that of in the non-exposed group of 28.97 (23.43–34.43) years (*Z* = 6.495, *P* < 0.001). The maternal weight of the HBsAg carrier group was 54.50 (44.00–72.46) kg; this was lower than that in the non-exposed group although the difference was not statistically significant 54.60 (43.20–73.80) kg (*Z* = 0.146, *P* > 0.05), as shown in Table 1.

**Table 1.** Demographic and clinical characteristics of cohort *n*

Variables	No HBsAg carrier	HBsAg carrier	*Z/x*^2^	*P*
	*n* = 18 693	*n* = 735		
Maternal age (years)	28.97 (23.43–34.43)	29.72 (23.94–34.91)	6.495	<0.001*
Maternal age (years)			22.712	<0.001*
<25	1509 (8.07)	45 (6.12)		
25–35	17 062 (91.28)	675 (91.84)		
≥35	122 (0.65)	15 (2.04)		
Maternal weight（kg）	54.60 (43.20–73.80)	54.50 (44.00–72.46)	0.146	0.884
Maternal weight（kg）			1.621	0.445
<50	4212 (22.53)	161 (21.91)		
50–70	13 542 (72.45)	544 (74.01)		
≥70	939 (5.02)	30 (4.08)		
GDM			2.762	0.097
No	16 734 (89.52)	672 (91.40)		
Yes	1959 (10.48)	63 (8.60)		
HDP			0.245	0.621
No	17 938 (95.96)	708 (96.33)		
Yes	755 (4.04)	27 (3.67)		
ICP			53.705	<0.001*
No	18 359 (98.22)	694 (94.42)		
Yes	334 (1.78)	41 (5.58)		
Thyroid dysfunction			3.400	0.065
No	16 193 (86.63)	654 (88.98)		
Yes	2500 (13.37)	81 (11.02)		
Hyperlipidaemia			2.698	0.100
No	17 814 (95.30)	710 (96.60)		
Yes	879 (4.70)	25 (3.40)		
Anaemia			0.013	0.908
No	13 617 (72.85)	534 (72.65)		
Yes	5076 (27.15)	201 (27.35)		
Thrombocytopenia			9.639	0.002**
No	18 539 (99.18)	721 (98.10)		
Yes	154 (0.82)	14 (1.90)		
Oligohydramnios			0.346	0.556
No	17 717 (94.78)	639 (94.29)		
Yes	976 (5.22)	42 (5.71)		
Caesarean scar			18.780	<0.001*
No	16 692 (89.30)	619 (84.22)		
Yes	2001 (10.70)	116 (15.78)		
Gestational infection			0.088	0.767
No	17 023 (91.07)	667 (90.75)		
Yes	1670 (8.93)	68 (9.25)		
Precious foetus			0.208	0.648
No	18 637 (99.70)	734 (99.86)		
Yes	56 (0.30)	1 (0.14)		
Postpartum haemorrhage			1.910	0.167
No	18 492 (98.92)	731 (99.46)		
Yes	201 (1.08)	4 (0.54)		
Uterine myoma			0.009	0.925
No	18 371 (98.28)	722 (98.23)		
Yes	322 (1.72)	13 (1.77)		
Placental abruption			0.001	0.975
No	18 606 (99.53)	731 (99.46)		
Yes	87 (0.47)	4 (0.54)		
Placenta previa			0.272	0.602
No	18 582 (99.41)	729 (99.18)		
Yes	111 (0.59)	6 (0.82)		
Pregnancy with hydronephrosis			0.164	0.341
No	18 670 (99.88)	735 (100)		
Yes	23 (0.12)	0 (0)		
Hypokalaemia			0.068	0.794
No	18 311 (97.96)	721 (98.10)		
Yes	382 (2.04)	14 (1.90)		
Umbilical cord torsion			0.316	0.574
No	18 572 (99.35)	732 (99.59)		
Yes	121 (0.65)	3 (0.41)		
Fallopian cysts			9.548	0.002**
No	18 440 (98.65)	715 (97.28)		
Yes	253 (1.35)	20 (2.72)		

GDM, gestational diabetes mellitus; HDP, hypertensive disorder pregnancy; ICP, intrahepatic cholestasis of pregnancy.

*P < 0.001; **P < 0.05.

### Univariate analysis of influencing factors

Univariate analysis showed that ICP, thrombocytopenia, caesarean scar, fallopian cysts, intrauterine distress, caesarean delivery and a low birth weight were significantly associated with an HBsAg carrier status (all *P* < 0.05). None of the other influencing factors showed any statistical significance when compared between the exposed group and the non-exposed group (all *P* > 0.05), as shown in Tables 1 and 2.

**Table 2.** Pregnancy outcomes of mothers with HBsAg-positve and negative group *n* (%)

Pregnancy outcomes	No HBsAg carrier	HBsAg carrier	*x*^2^	*P*
	*n* = 18 693	*n* = 735		
Retained placenta			1.406	0.236
No	18 595 (99.48)	734 (99.86)		
Yes	98 (0.52)	1 (0.14)		
Premature rupture of membranes			0.843	0.359
No	14 716 (78.72)	589 (80.14)		
Yes	3977 (21.28)	146 (19.86)		
Foetal distress			5.308	0.021**
No	17 246 (92.26)	661 (89.93)		
Yes	1447 (7.74)	74 (10.07)		
Cord entanglement			0.032	0.859
No	13 321 (71.26)	526 (71.56)		
Yes	5372 (28.74)	209 (28.44)		
Chorioamnionitis			0.192	0.661
No	18 212 (97.43)	718 (97.69)		
Yes	481 (2.57)	17 (2.31)		
Premature delivery			1.027	0.311
No	17 832 (95.39）	707 (96.19)		
Yes	861 (4.61）	28 (3.81）		
Caesarean delivery			10.549	0.001**
No	15 025 (80.38)	555 (75.51）		
Yes	3668 (19.62)	180 (24.49）		
Foetal live birth			3.214	0.073
No	1906 (10.20)	60 (8.16)		
Yes	16 787 (89.80)	675 (91.84)		
Foetal dystocia			0.563	0.453
No	17 717 (94.78)	692 (94.15)		
Yes	976 (5.22)	43 (5.85)		
Foetal stillbirth			0.051	0.821
No	18 446 (98.68)	726 (98.78)		
	247 (1.32)	9 (1.22)		
Macrosomia			1.085	0.298
No	17 970 (96.13)	701 (95.37)		
Yes	723 (3.87)	34 (4.63)		
Low birth weight			6.915	0.009**
No	18 576 (99.37)	724 (98.50)		
Yes	117 (0.63)	11 (1.50)		
Postpartum haemorrhage			0.382	0.537
No	17 786 (95.15)	703 (95.65)		
Yes	907 (4.85)	32 (4.35)		

*P < 0.001; **P < 0.05.

### Binary multivariate logistic regression analysis

After multivariate analyses were adjusted for maternal age, maternal weight, parity, caesarean history and abortion history, HBsAg-positive pregnant women had significantly higher incidences of adverse pregnancy outcomes, including ICP (aOR 3.169; 95% CI 2.266–4.432, *P* < 0.001), low birth weight (aOR 2.337; 95% CI 1.246–4.386, *P* = 0.008), thrombocytopenia (aOR 2.226; 95% CI 1.277–3.880, *P* = 0.005), fallopian cysts (aOR 1.610; 95% CI 1.002–2.586, *P* = 0.049), caesarean scar (CSP) (aOR 1.283; 95% CI 1.030–1.597, *P* = 0.026), foetal distress (aOR 1.414; 95% CI 1.102–1.813, *P* *=* 0.006), as shown in Table 3.

**Table 3.** Further binary logistic analysis of maternal characteristics and pregnancy outcomes

Indicators	*β*	SE	Wald	df	*P*	OR	95% CI for OR	*P*	aOR	95% CI for aOR
Maternal age	0.077	0.014	31.272	1	<0.001*	1.081	1.052–1.110	<0.001*	1.081	1.052–1.110
ICP	1.150	0.171	45.074	1	<0.001*	3.158	2.257–4.418	<0.001*	3.169	2.266–4.432
Thrombocytopenia	0.797	0.284	7.887	1	0.005**	2.218	1.272–3.867	0.005**	2.226	1.277–3.880
Caesarean scar	0.227	0.123	3.413	1	0.065	1.254	0.986–1.595	0.026**	1.283	1.030–1.597
Fallopian cysts	0.468	0.242	3.735	1	0.053	1.597	0.993–2.568	0.049**	1.610	1.002–2.586
Foetal distress	0.331	0.132	6.333	1	0.012**	1.392	1.076–1.802	0.006**	1.414	1.102–1.813
Caesarean delivery	0.045	0.101	0.203	1	0.652	1.046	0.859–1.275			
Low birth weight	0.845	0.321	6.915	1	0.009**	2.328	1.240–4.369	0.008**	2.337	1.246–4.386
Constant	−5.650	0.410	190.215	1	<0.001*	0.004				

OR, odds ratio; CI, confidence intervals; aOR, adjusted odds ratio; ICP, intrahepatic cholestasis of pregnancy.

*P < 0.001; **P < 0.05.

## Discussion

In this study, we investigated the outcomes of 735 pregnant women with HBsAg carrier status and 18 693 cases in a non-exposed group of subjects in Hangzhou, China. We found that the proportion of positive maternal HBsAg carrier was 3.78%; this was slightly higher than the 3.3% rate reported previously in Kunming, China [18].

In China, people such as medical staff, nursery staff, patients receiving organ transplants, people frequently accessible to blood transfusions, blood products or intravenous drug, people with poor immunity or vulnerable to trauma, people with multiple sexual partners or with family members of HBsAg-positive, etc., are under high risk of hepatitis B [19]. Furthermore, we found that the maternal age of the HBsAg carrier status was slightly higher than in the non-exposed group (aOR = 1.081); this was generally consistent with previous reports in the related literature [20, 21].

We also found that pregnant women in the HBsAg carrier group had a higher risk of developing ICP (aOR = 3.169). The results of a previous meta-analysis by Jiang *et al*. [22] showed that pregnant women infected with HBV had a higher risk of developing ICP and that ICP patients had an increased risk of HBV infection. In another study, Xiong *et al*. [23] suggested that HBV-infected pregnant women (HBsAg-positive or HBsAg- and HBeAg-positive) may have an increased risk of ICP. Cai *et al*. [24] also showed that chronic HBV infection during pregnancy may increase the risk of ICP, PROM and large for gestational age pregnancies. Collectively, these studies indicated that ICP was a high risk factor for HBsAg carrier and that the diagnosis and treatment of ICP in HBsAg carrier should be strengthened in the clinic.

The risk of developing thrombocytopenia (aOR = 2.226) was higher among pregnant women in the HBsAg carrier group, as shown in Table 3. In a previous large cohort study of an apparently healthy population, HBsAg positivity was strongly associated with thrombocytopenia, thus indicating that mechanisms associated with thrombocytopenia other than portal hypertension may exist in healthy HBV carriers [25]. Immature platelet fraction (IPF%) is known to be higher during the course of thrombocytopenia, thus indicating that platelet destruction/sequestration caused by hypersplenism was the main factor underlying the thrombocytopenia observed in patients with hepatitis B virus-related chronic hepatitis (CHB) [26].

Our analysis also identified an increased risk of fallopian cysts (aOR = 1.610) and caesarean scars (aOR = 1.283) in pregnant women in the HBsAg-positive group. Furthermore, cases involving caesarean delivery that are complicated by adhesions attributable to a previous caesarean delivery are known to be associated with an increased risk of peri- and immediate post-partum infectious morbidity [27]. Repeat caesarean delivery is known to increase perioperative risks, including adhesions, infections, wound complications and bleeding [28]. In addition to increasing the risk of developing infectious diseases, other studies have investigated whether adhesions caused by tumours, or following caesarean delivery, could increase the probability of HBV infection. For example, Xiao *et al*. found that in a hepatitis B infection group, the incidence of neonatal distress and asphyxia was significantly higher than that in a control group [29]. In another study, Sirilert [30] showed that the incidence of low birth weight was significantly higher among women with a positive HBeAg status relative risk (1.258, 95% CI 1.053–1.505); these results were consistent with those described in the present study.

Several studies have reported an increased risk of GDM in pregnant women who are HBsAg carrier. For example, Giles *et al*. showed that HBV was associated with GDM with a risk ratio of 1.750 for GDM (95% CI 1.600–1.900) [31]. The highest incidence (37.10%) of GDM was observed in women with HBV and a body mass index of >40 Kg/m^2^. Another study showed that HBsAg-positive women in Xiamen, China, had a higher risk of GDM and caesarean delivery [20]. In contrast, we found that pregnant women in the HBsAg carrier group were not associated with the risk of developing GDM.

We investigated the relationship between an HBsAg carrier status and pregnancy outcome in Hangzhou. Although our study included a relatively large sample size, there were some limitations that need to be considered. First, our study involved a relatively large sample size; however, our findings were only representative of the Hangzhou region of China. Second, our study lacked information related to other antigens and antibodies (HBsAb, HBeAg, HBeAb, HBcAb) for hepatitis B, and liver function. Furthermore, our study lacked information relating to the effect of different combinations of HBV-related states on maternal outcomes. Future studies should include longer follow-up periods, a greater number of variables and larger sample sizes.

In conclusion, in Hangzhou, China, the prevalence of maternal HBsAg carrier was 3.78%. Our analysis also showed that pregnant women who were HBsAg carrier were at a higher risk of adverse pregnancy outcomes, including ICP, low birth weight, thrombocytopenia, fallopian cysts, caesarean scar, foetal distress, in HBsAg carrier pregnant women.

## Availability of data and materials

The datasets used and/or analysed during the current study are available from the corresponding author on reasonable request.
